# The interface with naturopathy in rural primary health care: a survey of referral practices of general practitioners in rural and regional New South Wales, Australia

**DOI:** 10.1186/1472-6882-14-238

**Published:** 2014-07-11

**Authors:** Jon L Wardle, David W Sibbritt, Jon Adams

**Affiliations:** 1 Australian Research Centre in Complementary and Integrative Medicine (ARCCIM), Faculty of Health, University of Technology Sydney, 235-253 Jones St, Ultimo, NSW 2007, Australia

**Keywords:** Naturopathy, Complementary medicine, General practice, Rural healthcare, Health services, Referral, Interdisciplinary care, Primary care

## Abstract

**Background:**

Naturopathy forms an increasingly significant part of the Australian healthcare setting, with high utilisation of naturopaths by the Australian public and a large therapeutic footprint in rural and regional Australia. However, despite these circumstances, there has been little exploration of the interface between naturopathy providers and conventional primary health care practitioners in rural and regional Australia. The study reported here examined the referral practices and factors that underlie referral to naturopaths amongst a sample of rural and regional Australian general practitioners (GPs).

**Methods:**

A 27-item questionnaire was sent to all 1,486 GPs currently practising in rural and regional Divisions of General Practice in New South Wales, Australia.

**Results:**

A total of 585 GPs responded to the questionnaire, with 49 questionnaires returned as "no longer at this address" (response rate: 40.7%). One-quarter of GPs (25.8%) referred to a naturopath at least a few times per year while nearly half (48.8%) stated that they would not refer to a naturopath under any circumstances. GPs were more likely to refer to a naturopath if the GP: was not initially from a rural area (OR=1.78; 95% CI: 0.95, 3.33); believes in the efficacy of naturopathy (OR=5.62; 95% CI: 2.42, 11.36); has seen positive results from naturopathy previously (OR=2.61; 95% CI: 1.35, 5.05); perceives a lack of other treatment options for their patient (OR=5.25; 95% CI: 2.42; 11.36); uses peer-reviewed literature as their major source of CAM information (OR=3.03; 95% CI: 1.65, 5.55); uses CAM practitioners as a major source of CAM information (OR=6.09; 95% CI: 2.91, 12.72); and does not have an existing relationship with any CAM provider (OR=3.03; 95% CI: 1.53, 6.25).

**Conclusions:**

There is little interaction (both via referrals as well as the development of professional relationships) between the naturopathic and GP communities in rural and regional Australia, with significant levels of both support and opposition for naturopathic referral amongst GPs. The significant presence and high utilisation of naturopathy in rural primary health care, which appears to work in parallel to conventional medical care rather than in conjunction with it, should serve as an impetus for increased research into naturopathy practice, policy and regulation in rural and regional health.

## Background

Complementary and alternative medicines (CAM) – a diverse group of health care practices and products not considered part of conventional medicine [1] – are used in some form by an increasing proportion of Australians. Australian studies have suggested that visits to CAM practitioners may account for up to half of all health consultations and that Australians have more out-of-pocket expenditure on CAM than conventional medicine [2]. In some regions of Australia, CAM practitioners may be present in numbers equal to conventional primary care providers [3].

Naturopathy is Australia’s largest CAM profession [4], and plays an increasingly role in the Australian healthcare system. National studies suggest that over 10% of women regularly consult with a naturopath or herbalist [5], increasing to over 15% in complex conditions such as cancer [6], and many naturopaths are used as primary care providers [7]. Naturopaths form nearly half of the ‘primary-care capable’ non-medical CAM workforce in rural and regional^a^ Australia [3]. Naturopathy as a professional group are experiencing substantial growth in Australia, though lack of regulation means that training and practice standards remain variable, with possible repercussions for public health and safety [8].

Naturopathic medicine is defined not by the therapies, practices or substances used, but rather the principles that underlie and determine its practice. These include the following: supporting the healing power of nature; finding the root cause of ill health, first doing no harm, treating the whole person rather than individual disease processes, prevention and doctor as teacher [9]. Unlike many other CAM professions in Australia, the profession of naturopathy remains fragmented, with over 90 associations purporting to represent naturopaths [10]. This fragmentation, combined with the variability of standards in training and practice, has somewhat hampered the professional development of naturopathy due to professional infighting and the nefarious influence of vested interests [11].

Despite sustained growth of the naturopathic profession in Australia, both in terms of practitioner numbers and public utilisation, there has been relatively little research focused on this practitioner group, and how it interacts with primary health care.

General practice is one branch of medicine where CAM has long made an impact [12,13], with data from Australian studies demonstrating considerable levels of referral from general practitioners (GPs) to CAM practitioners [14-16]. However, such integration is an evolving and sometimes controversial phenomenon. There also appears to be a preference amongst GPs for referral to other GPs practising CAM therapies, rather than referral to non-medically trained CAM providers, such as naturopaths [16]. There is, however, also evidence of closer working relationships developing between non-medical CAM practitioners and GPs [14,17]. However, there remains uneven integration of CAMs amongst GPs, with individual CAM therapies and practitioner types attract varying levels of support [14,18].

Previous studies have indicated low levels of interaction between GPs and naturopaths in Australia. A national workforce survey of naturopaths and herbalists found that naturopaths referred 7% of their patients to a general medical practitioner, and that 4% of their patients were the result of referral by a GP [10]. National surveys of GPs have indicated that 22-44% of GPs have referred to a naturopath at least a few times in the past year [14,15].

There also appears to be substantial levels of opposition to naturopathy amongst the Australian GP community. In one national survey, 71% of GPs stated that they thought that naturopathy was seldom or not effective, and that 26% would actively discourage a patient’s suggestion that they attend a naturopathic consultation [14]. Such opposition could be related to the unregulated nature of the profession, as 73% thought that naturopathic practitioners needed to be regulated by the government [14]. Such opposition could also be due to philosophical or ideological opposition to naturopathic ‘whole practice’, which has been described in the Australian medical literature as pseudoscientific and incompatible with conventional medical principles [19].

There also appears to be discordance between medical practitioner support of individual therapies and practises used by naturopaths, and their support of naturopaths. For example, a national survey found that whilst medical practitioner perceptions of efficacy for vitamin and mineral therapy and naturopathy were similar, significantly more medical practitioners would consider receiving vitamin and mineral therapy than naturopathic treatment [14]. Such dissonance between therapy and practitioner support may reflect philosophical or ideological tensions, which has also been observed in other CAM. For example, although there appears to be high utilisation and support of acupuncture amongst the Australian GP community [20], there appears to be little support and interaction with Chinese medicine practitioners [21].

Research has also highlighted increased use of CAM by rural populations when compared to urban populations, both in Australia and internationally [22,23]. However, the only Australian study to explore this did not find significant differences between utilisation of naturopathy in rural versus urban populations [5]. Qualitative exploration of naturopathic practitioners in Australia has, however, suggested that practitioners perceive a greater demand for their services in rural areas, and that they are expected to have a larger primary care role than practitioners in urban areas [7]. Moreover, naturopaths do form the largest CAM professional group in rural and regional areas of Australia [3].

The substantial therapeutic footprint of naturopathy in the Australian healthcare sector has implications for general practice and healthcare delivery in rural and regional Australia. However, despite the extensive presence of naturopathic practitioners in rural and regional Australia, and the high levels of utilisation amongst the population in these regions, there has been little research to date exploring the level of integration or the factors that underlie any integration of naturopathy in rural and regional general practice in Australia. This paper provides a first step in addressing this research gap by exploring practise and referral patterns of GPs in relation to naturopaths in rural and regional areas of Australia’s largest state (New South Wales).

## Methods

A 27-item questionnaire was mailed by post to all 1,486 GPs registered as practising in rural and regional General Practice Divisions of NSW, with a reminder card sent after two months (see Additional file 1). The questionnaire was adapted for rural and regional use from previous Australian surveys of GP attitudes, use and practices of CAM [14,15]. The survey was piloted at the Department of General Practice, School of Medicine and Public Health, University of Newcastle, with modifications made based on feedback to ensure the instrument was clinically relevant. GPs were asked about their knowledge, attitudes, and practice and referral patterns to naturopaths, and about CAM use in their areas more generally.

The final survey questionnaire contained 27 items which included multiple choice and multiple response close ended questions. This paper reports referral to naturopathy; analyses of referral to other professions has been reported previously [20-22]. The survey had five general areas: the GPs’ assumptions on naturopath visits by patients in their area; the GPs’ personal use and knowledge of naturopathy; the GPs’ professional relationship with naturopathy practice and naturopathic practitioners; the GPs’ information seeking behaviours on naturopathy; and the GPs’ specific opinions on naturopathy. GPs were also asked for demographic and practice information such as gender, age, number of years in practice, location of practice, number of patients seen per week and country of graduation. The survey questionnaire was accompanied with an information sheet, and a statement was present on the survey questionnaire highlighting that completion of the questionnaire constituted informed consent. A postage paid reply envelope was provided to allow for questionnaires (which contained no identifying information) to be sent back anonymously. Ethical approval for the study was obtained from the School of Population Health Research Ethics Committee of the University of Queensland (JW130508) and the Human Research Ethics Committee of the University of Newcastle (H-2008-0344).

Rural and regional areas were defined by their classification in the Rural, Remote and Metropolitan Area (RRMA) classifications [24]. The RRMA classification categorises areas based on population and remoteness as large or small metropolitan (1–2), large, small and other rural centres (3–5); and remote or other remote (6–7). To minimise the effects of local variation, every rural and regional GP in Australia’s largest state (New South Wales) was surveyed.

Questionnaire data was analysed using descriptive statistics via frequency distributions and cross-tabulations. Demographic and practice characteristics of GPs who referred to a naturopath often (at least monthly) and seldom or never were compared using chi-square tests. Logistic regression modelling, that included all practitioner and practice characteristics variables, was conducted using a backwards stepwise method of elimination using a likelihood ratio test, to parsimoniously predict referral to naturopaths. Statistical significance was set at the α = 0.05 level. All data were examined for missing data and outliers, and cleaned prior to data analysis. Data were analysed using the software program STATA 11.

## Results

A total of 585 questionnaires were returned completed, with 49 questionnaires returned uncompleted as ‘no longer at this address’; giving a response rate of 40.7%. Respondents had an average age between 45 and 54 years and were 53.5% male. Over three-quarters of respondents (77.8%, n = 456) had completed their medical training at an Australian university. Apart from a slight over-representation of women in the study sample, the respondent profile was broadly representative of the GP community in the study area in relation to average age and training location [25].

The referral rates of rural and regional GPs to naturopaths are shown in Table 1. Approximately one-in-ten (10.1%, n = 59) GPs referred to a naturopath at least once per month, with a further 15.7% (n = 92) referring a few times per year. There was substantial opposition to referral to naturopaths in rural general practice, with 48.8% (n = 286) stating that they would not refer their patients to a naturopath under any circumstances. Most GPs were aware of naturopaths practising in their local area, with only 5.3% (n = 31) of respondents unable to identify naturopaths to refer their patients to.

**Table 1:** Referral rates of rural GPs to naturopaths in the past 12 months (n = 585)

**Referral rates**	**Frequency (Percent)**
At least weekly	24 (4.1%)
At least monthly	35 (6.0%)
A few times per year	92 (15.7%)
I have not referred but would consider	114 (19.5%)
I would never refer	286 (48.8%)
I do not know of any practitioners	31 (5.3%)
No response	3 (0.5%)

Some GPs in this study also self-identified as naturopathic practitioners themselves, with 1.9% (n = 11) stating that they had practised naturopathy during the past 12 months (data not shown). A small number of GPs (7.3%; n = 43) had a personal professional relationship with a specific individual naturopath to whom they referred their patients.

Table 2 shows a comparison between GPs who referred to a naturopath often (at least weekly or at least monthly) and seldom (less than a few times per year or never) by demographic characteristics. GPs were significantly more likely to refer to a naturopath if they had lower (under 100 patients per week) or high (over 151 patients per week) (p < 0.001). There was also a significant association with age of GP and referral to a naturopath (p = 0.011), though with no consistent pattern. GPs were significantly more likely to refer to a naturopath if they were aged between 35 and 44. There were no significant associations between referral to a naturopath and sex, level of rurality of GP practice and country of graduation from medical school.

**Table 2:** Demographic and practice characteristics associated with referral to a naturopath by rural and regional GPs in New South Wales, Australia (n = 585*)

	**Referral to naturopathy**		
**Demographic characteristics**	**Weekly or monthly**	**Seldom or never**	**p-value**
	**%**	**%**	
Sex			
Male	48.1	55.4	0.115
Female	51.9	44.6	
Age			
25-34	3.9	10.7	0.011
35-44	25.3	20.2	
45-54	37.7	37.8	
55-64	29.3	23.2	
>65	3.9	8.1	
RRMA			
3	33.8	26.5	0.067
4	39.0	42.7	
5	22.1	26.2	
6	3.9	2.3	
7	1.3	2.3	
Australian graduate?			
Yes	78.6	77.7	0.828
No	21.4	22.3	
Initially from a rural area?			
Yes	26.0	34.6	0.051
No	74.0	65.4	
Patient load (per week)			
<50	21.4	14.2	<0.001
51-100	37.0	35.8	
101-150	14.3	35.3	
151-200	20.8	9.7	
>200	6.5	5.1	

Table 3 shows a comparison between GPs who referred to a naturopath often (at least weekly or at least monthly) and seldom (less than a few times per year or never) by other factors. Referral to a naturopath was significantly associated with level of knowledge about naturopathy (p < 0.001), the number of patients asking about CAM (p < 0.001), personal CAM use by the GP (p < 0.001), not having other options available (p = 0.010), having had positive results with naturopaths previously (p < 0.001), using CAM practitioners as a major source for CAM information (p < 0.001), belief in the efficacy of naturopathy (p < 0.001), being interested in increasing CAM knowledge (p < 0.001) having prescribed CAM previously to patients (p < 0.001) and being comfortable with referral to a naturopath (p < 0.001).

**Table 3:** Other factors associated with referral to a naturopath by rural and regional GPs in New South Wales, Australia (n = 585)

		**Referral to naturopathy**		
**Factors**		**Weekly or monthly**	**Seldom or never**	**p-value**
		**%**	**%**	
Level of knowledge				
	Excellent	13.6	3.3	<0.001
	Very Good	15.6	6.0	
	Satisfactory	39.6	28.8	
	Poor	31.2	49.4	
	Very Poor	0.0	12.5	
Patients asked about CAM				
	<10%	25.3	39.0	<0.001
	11-25%	29.9	46.9	
	26-50%	11.7	7.0	
	>50%	33.1	7.2	
Personal use				
	Regularly	40.3	3.0	<0.001
	Often	17.5	17.9	
	Once/Rarely	21.4	34.1	
	Never, but would consider	14.9	12.3	
	Never, and would not consider	5.8	31.1	
Access to medical specialists is a problem				
	Yes	5.8	1.6	0.010
	No	94.2	98.4	
Patient request for referral				
	Yes	59.7	55.2	0.332
	No	40.3	44.8	
Lack of other options				
	Yes	20.1	8.1	<0.001
	No	79.9	91.9	
Positive results previously				
	Yes	75.3	39.0	<0.001
	No	24.7	61.0	
Information from CAM practitioner?				
	Yes	37.0	13.2	<0.001
	No	63.0	86.8	
Information from patients?				
	Yes	46.1	47.6	0.755
	No	53.9	52.4	
Belief in efficacy				
	Yes	68.8	17.4	<0.001
	No	31.2	82.6	
Interested in increasing CAM knowledge?				
	Yes	73.4	48.8	<0.001
	No	26.6	51.2	
Have prescribed CAM to patients				
	Yes	85.7	67.0	<0.001
	No	14.3	33.0	
Comfort level				
	Comfortable in general	31.8	2.6	<0.001
	Only in specific circumstances	45.3	10.9	
	Only if I knew them in person	18.2	21.6	
	I would not refer	4.7	65.0	

### Predictive factors

The result of multiple logistic regression modelling to determine the most important predictive factors for referring to naturopaths is shown in Table 4. This modelling shows that GPs not initially from a rural area were 1.78 (95% CI: 0.95, 3.33) times more likely to refer to a naturopath at least once per month than those who were initially from a rural area. GPs who believed in the efficacy of naturopathy were 5.62 (95% CI: 2.42, 11.36) times more likely to refer to a naturopath at least once per month than those who did not believe in the efficacy of naturopathy. GPs who had seen positive results from naturopathy previously were 2.61 (95% CI: 1.35, 5.05) times more likely to refer to a naturopath at least once per month than those who had not seen positive results. GPs were 5.25 (95% CI: 2.42, 11.36) more likely to refer to a naturopath at least once per month if they perceived there were no other options available. Information sources also affected referral, with GPs who identified peer-reviewed literature as a major source of CAM information being 3.03 (95% CI: 1.65, 5.55) times more likely to refer to a naturopath at least once per month than those who did not and GPs who identified CAM practitioners as a major source of CAM information being 6.09 (95% CI: 2.91, 12.72) times more likely to refer to a naturopath at least once per month than those who did not. GPs who *did not* have an existing professional relationship with a CAM practitioner were 3.03 (95% CI: 1.53, 6.25) times as likely to refer to a naturopath more than once per month than those who had a pre-existing relationship with another CAM practitioner.

**Table 4:** Predictive factors for referral by GPs to naturopaths at least once per month by rural and regional GPs in New South Wales, Australia (n = 573)

**Factor**	**Odds ratio**	**95% CI**
Originally from a rural area?		
No	1.00	─
Yes	0.56	0.30, 1.05
Lack of other options		
No	1.00	─
Yes	5.25	2.42, 11.36
Positive results previously		
No	1.00	─
Yes	2.61	1.35, 5.05
Belief in efficacy		
No	1.00	─
Yes	5.62	3.01, 10.49
Peer-reviewed literature a major source of information		
No	1.00	─
Yes	3.03	1.65, 5.55
CAM practitioners a major source of information		
No	1.00	─
Yes	6.09	2.91, 12.72
Professional relationship with CAM provider		
No	1.00	─
Yes	0.33	0.16, 0.65

## Discussion

To our knowledge, this is the first focused examination of GP referral to naturopaths in rural and regional Australia. Our study findings show that despite the high presence of naturopaths in rural and regional Australia [3], there is a relatively low GP-directed interaction between naturopaths and rural GPs. The finding that just over one-quarter of GPs in this study had referred to a naturopath at least once over the past few years does fit within the lower levels of referral observed in previous national studies [14,15].

The relatively low level of referral to naturopaths occurred even though GPs were more able to identify naturopaths than other CAM practitioners in rural areas. Nearly half of all GPs in this study stated that they would refuse to refer to a naturopath under any circumstances. The finding observed in this study indicates that professional tensions may exist between the two groups. This could be the result of ideological or philosophical conflict between the two professions, as the validity of naturopathic practice has been recently questioned in high-profile Australian medical literature, with some commentators suggesting their unscientific nature means that further research or practice of the discipline needs to be actively discouraged [19].

This opposition could also be partly due to naturopaths competing more directly with GPs in rural communities than other CAM practitioners. The broad scope, eclectic and philosophically-defined nature of naturopathic practice has meant that naturopathic practitioners have been described as the ‘general practitioners of CAM’ [9,26]. This differs from many other CAM practitioners, who may have a more clearly delineated scope (for example, acupuncturists may be defined by their tool of trade, chiropractors and osteopaths by their focus on musculoskeletal treatment). As such, naturopaths may be viewed as a more direct ‘alternative’ competitor in a medical pluralistic model of care, rather than a ‘complementary’ adjuvant therapy that can be incorporated into primary practice.

The broad scope nature of naturopathy may also partly explain why a GP having a professional relationship with any other CAM provider negatively predicted referral to a naturopath, as the broad scope of naturopathy would mean that elements of their practitioner role could be more easily assumed by other provider types than CAM practitioners with more restricted scopes of practice. The fact that high levels of GP support for major elements of naturopathic practice, such as herbal medicine and nutritional and vitamin therapy, have not translated to high levels of support or referral to naturopaths would appear to support the notion that professional tensions exist between these two groups. Such tensions may suggest that opposition is related more to perceived risks associated with the practice behaviours of naturopaths, such as perceived conflict of interest in product sales [27] or variable training [8], than they are to specific elements of naturopathic practice. Given the recent high-profile attacks on CAM practitioners garnered in the Australian medical media [19], further examination of differences in perception and attitudes of GPs to medical and non-medical practice of CAM may offer valuable insights into how these therapies are integrated in primary health care.

One of the more interesting results of this study was the finding that GPs who use peer-reviewed literature as their major source of information about CAM were three times more likely to refer to a naturopath than those who focused on other information sources. Although Australian biomedical journals have covered CAM topics regularly, articles have focused on issues of risk rather than efficacy [28]. Additionally, studies have indicated that press coverage of clinical trials of CAM may focus on negative aspects, whilst conventional treatment coverage may focus on positive aspects of treatment [29]. GPs wanting to explore the efficacy of various CAM objectively may need to venture further into the peer-reviewed literature, where there may be evidence of efficacy for specific elements of naturopathic medicine [9].

The broad scope of naturopathic practice may be a potential advantage for eliciting GP referrals for CAM, as the ability to practise a broad range of CAM therapies means that evidence for non-naturopathic CAM could also translate into referral. This could also partly explain why the use of peer-reviewed literature appears to be predictive of referral to naturopathy, but negatively predicts referral to specific therapy-based CAM practitioners such as homeopaths [30]. This result does suggest that the information sources do have an impact on GP referral patterns to CAM providers, and further investigation of how conventional medical providers use information sources for their CAM information, and how this affects their practice, is further warranted.

The rural nature of this sample in this study could affect the professional relationships with individual practitioners and predict referral, as smaller communities may facilitate increased interaction between CAM and conventional providers [22,31,32]. This could in turn facilitate increased levels of referrals by rural and regional GPs to CAM providers (such as naturopaths) compared to their urban counterparts, even if there is little support for specific professions such as naturopathy. This may explain why both a patient having few other treatment options may independently predict referral to naturopaths in this study. Similarly, smaller communities may also facilitate communication of positive CAM results, which may explain why positive results with naturopathy independently predicted referral to naturopaths. These factors could, in turn, be potentially positive or potentially negative for naturopaths, as even though referral to naturopaths does appear to be negatively affected by the presence of other CAM provides, naturopaths may be partly insulated by these factors given they are the largest CAM profession in rural Australia, and may be present in locations where there are no other CAM providers [3]. Further investigation into the effects community factors have on CAM use and integration is warranted, as is further investigation of how communities navigate and interface between different CAM provider types.

Though limited to Australia’s largest state (by population), the large and varied study area was chosen to be broadly representative of Australian rural and regional general practice demographics. Nevertheless, the demographics of the GPs in this study compared to broader national general practice demographic (being as they are drawn from rural and regional areas and exhibiting a higher proportion of females [25]) should be considered when generalising the study’s results to the broader Australian general practice population. Additionally, the results of this study need to be viewed in the context that referral (or lack of referral) does not in-and-of itself measure or imply effectiveness of care.

Other limitations of the study, which are common amongst other cross-sectional studies that utilise questionnaires, include the use of self-reported data and possible recall bias inherent in retrospective collection of data over a 12 month period, as well as self-selection which may have resulted in a response bias. Response bias could also have resulted as CAM is often a controversial issue in medical practice, and those GPs with particular strong views may have been more likely to participate. Note that the response rate is typical for large-scale GP surveys on CAM conducted in Australia over the past decade, which have reported response rates of between 29-58% [14,18,33], as well as with general surveys of Australian GPs, which routinely have difficulty receiving response rates of over 30% [34].

## Conclusions

Our study reveals low levels of interaction (both via referrals as well as the development of professional relationships) between the GP and naturopathic communities in rural and regional Australia. This contrasts with the high use of naturopathy in the Australian community, which when combined with a high presence in rural and regional Australia highlights the important need for more research in this area, to ascertain the impact this profession has upon patient care delivery.

The substantial presence and high utilisation naturopathy in rural primary health care, which appears to work in parallel to conventional medical care rather than in conjunction with it, should serve as an impetus for increased research into naturopathy practice, policy and regulation in rural and regional health.

## Endnote

^a^The official terminology ‘rural and regional’ is used in the Australian setting to reflect varying degrees of rurality. In this sense, ‘rural and regional’ is interchangeable with ‘rural’.

## Competing interests

The authors declare they have no competing interests.

## Authors’ contributions

JW was involved with conception and design of the study, collection of the data, analysing and interpreting the data and drafting and revising the manuscript; DS was involved with conception and design of the study, analysing and interpreting the data and drafting and revising the manuscript; JA was involved with conception and design of the study, analysing and interpreting the data and drafting and revising the manuscript. All authors read and approved the final manuscript.

## Pre-publication history

The pre-publication history for this paper can be accessed here:

http://www.biomedcentral.com/1472-6882/14/238/prepub

## Supplementary Material

Additional file 1 Survey of Rural GP attitudes and perceptions of CAM. Click here for file
